# Enhanced Medial Prefrontal Cortex and Hippocampal Activity Improves Memory Generalization in APP/PS1 Mice: A Multimodal Animal MRI Study

**DOI:** 10.3389/fncel.2022.848967

**Published:** 2022-03-21

**Authors:** Weilin Liu, Jianhong Li, Le Li, Yuhao Zhang, Minguang Yang, Shengxiang Liang, Long Li, Yaling Dai, Lewen Chen, Weiwei Jia, Xiaojun He, Huawei Lin, Jing Tao

**Affiliations:** ^1^National-Local Joint Engineering Research Center of Rehabilitation Medicine Technology, Fujian University of Traditional Chinese Medicine, Fuzhou, China; ^2^TCM Rehabilitation Research Center of SATCM, Fujian University of Traditional Chinese Medicine, Fuzhou, China; ^3^College of Rehabilitation Medicine, Fujian University of Traditional Chinese Medicine, Fuzhou, China

**Keywords:** Alzheimer’s disease, cognitive training, generalization, functional activity, neurochemical metabolism

## Abstract

Memory generalization allows individuals to extend previously learned movement patterns to similar environments, contributing to cognitive flexibility. In Alzheimer’s disease (AD), the disturbance of generalization is responsible for the deficits of episodic memory, causing patients with AD to forget or misplace things, even lose track of the way home. Cognitive training can effectively improve the cognition of patients with AD through changing thinking mode and memory flexibility. In this study, a T-shaped maze was utilized to simulate cognitive training in APP/PS1 mice to elucidate the potential mechanisms of beneficial effects after cognitive training. We found that cognitive training conducted by a T-shaped maze for 4 weeks can improve the memory generalization ability of APP/PS1 mice. The results of functional magnetic resonance imaging (fMRI) showed that the functional activity of the medial prefrontal cortex (mPFC) and hippocampus was enhanced after cognitive training, and the results of magnetic resonance spectroscopy (MRS) showed that the neurochemical metabolism of N-acetyl aspartate (NAA) and glutamic acid (Glu) in mPFC, hippocampus and reuniens (Re) thalamic nucleus were escalated. Furthermore, the functional activity of mPFC and hippocampus was negatively correlated with the escape latency in memory generalization test. Therefore, these results suggested that cognitive training might improve memory generalization through enhancing the functional activity of mPFC and hippocampus and increasing the metabolism of NAA and Glu in the brain regions of mPFC, hippocampus and Re nucleus.

## Introduction

Memory generalization allows individuals to extend previously learned movement patterns to similar environments, which contributes to the maintenance of normal life activities. Studies in recent years have indicated that the disturbance of generalization is linked to multiple neurological and psychiatric diseases, such as posttraumatic stress disorder (PTSD) (Pedraza et al., 2019; Wang et al., 2021). Even though overgeneralization of fear memories is linked to pathological states, generalization plays an essential role in the processes of learning and memory. People with Alzheimer’s disease (AD) typically experience deficits in memory, executive function, language, and attention. Of these dysfunctions, episodic memory impairment has been widely recognized as the most noticeable and earliest symptoms, and the impairment of episodic memory causes patients with AD to forget or misplace things, experience difficulty in recalling the details of recent events, and lose track of the way home, disrupting the quality of normal life (Winocur et al., 2001). The improvement of generalization ability may help in the maintenance of normal episodic memory.

Based upon previous literature, the cognition of patients with AD can be improved after many forms of cognitive training, and the beneficial effects from cognitive training are associated with changed thinking mode and improved memory flexibility (Markovic et al., 2020). Previous studies have showed that computer-assisted cognitive training can effectively improve the learning memory ability in patients with mild cognitive impairment or Alzheimer’s disease (Herrera et al., 2012; Vermeij et al., 2016; Cavallo and Angilletta, 2017). A clinical randomized controlled trial study demonstrated that memory training can enhance memory performance in older adults (Ball et al., 2002). A recent study also indicated that people with mild to moderate AD can learn and recall new episodic information well through memory training (Small and Cochrane, 2020). Since generalization ability is highly important for normal memory function, we can guess that cognitive training might improve memory capacity through memory generalization ability. In this study, we utilized a T-shaped maze to train memory flexibility in APP/PS1 mice to explore the mechanism for improved generalization ability after cognition training.

In the central nervous system (CNS), the hippocampus is a critical brain region that regulates memory and learning ability, especially episodic memories (Hainmueller and Bartos, 2020). The medial prefrontal cortex (mPFC) plays an important regulatory role in executive functions, strategy switching, and behavioral flexibility (Ragozzino et al., 2003; Ghods-Sharifi et al., 2008). The bidirectional synaptic connections among reuniens (Re) nucleus, mPFC and hippocampus have been confirmed with the application of virus tracing technology in recent years (Xu and Sudhof, 2013; Varela et al., 2014). In the process of learning and memory, the neural circuit of mPFC-Re-hippocampus coordinates the information between mPFC and hippocampus regulating memory generalization (Xu and Sudhof, 2013; Ito et al., 2018). In addition, the memory integration mechanism between mPFC and hippocampus supports not only the generalization of episodic memory but also conceptual learning. Studies pointed out that memory impairment in the early stage of patients with AD positively correlated with the atrophy of the prefrontal cortex and hippocampus (Carter et al., 2014). The neural activity of the Re nucleus can be influenced by tetanus toxin (TetTox) and lentiviral technology. Using these technologies, studies found that inhibited neural signal transmission in the Re nucleus would reduce the expression of c-fos in the CA1 regions of the hippocampus and mPFC, restraining memory generalization (Varela et al., 2014; Ito et al., 2015, 2018).

The destruction of the functional connection of the mPFC-Re-hippocampus neural circuit in patients with AD is associated with the decline of memory generalization ability. Previous studies showed that cognitive training could improve cognitive function through enhancing the functional connections between the frontal cortex, thalamus, and hippocampus (Li et al., 2016). Based on this, in this study, we utilized a T-shaped maze to simulate cognitive training to enhance the memory flexibility of APP/PS1 mice. Then, fMRI and MRS technologies were combined to elucidate the potential mechanisms of beneficial effects after cognitive training.

## Materials and Methods

### Animals and Ethics

Male APP/PS1 double-transgenic mice [C57BL/6 × C3H] were purchased from the Nanjing Biomedical Research Institute of the Nanjing University as AD model mice. The mice required for the experiment were obtained by crossing male APP/PS1 double-transgenic mice with female wild-type mice [C57BL/6]. The animals were raised in SPF-level animal experiment center of the Fujian University of Traditional Chinese Medicine (FJTCM). Each cage contains 4 mice, and all animals were permitted to food and water. The environment of the SPF room is kept constant, with a standard day and night system (12 h light + 12 h dark), the room temperature at about 22°C, and the humidity of 50–70%. The experiments were operated and implemented in strict accordance with the provisions of the national animal protection laws and regulations, and all the ethics of experiments have been endorsed by the Animal Management Committee of the Fujian University of Traditional Chinese Medicine (FJTCMIACUC 2019031).

### Experimental Protocol

**FIGURE 1:**
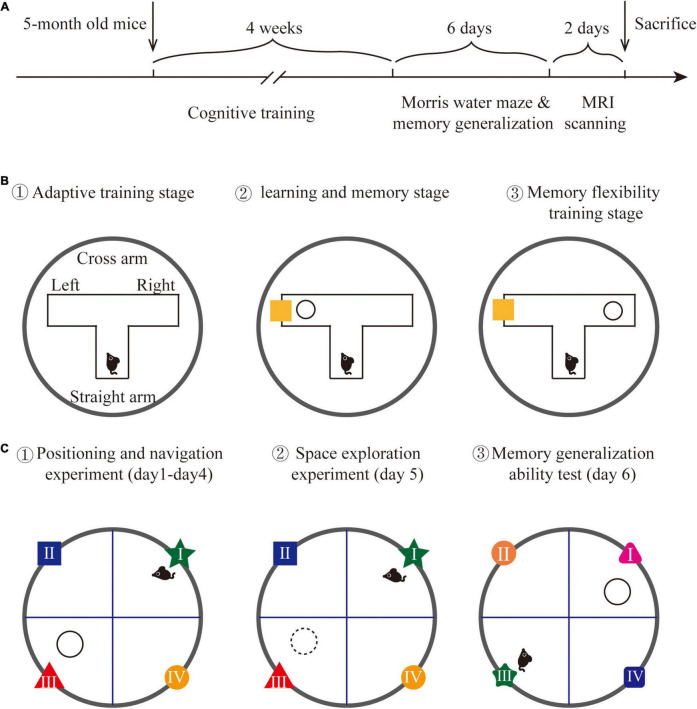
Experimental design. **(A)** The detailed time line of this experiment. **(B)** The schematic graph of water T-maze cognitive training. **(C)** The schematic graph of the Morris water maze and memory generalization test.

The PCR technology was applied to identify the genotype of each mouse. According to previous studies, APP/PS1 mice present significant memory generalization decline at 6 months. Thus, this experiment selected 5-month-old APP/PS1 mice conducting cognitive training for 4 weeks. According to the random digital table method, the APP/PS1 double-transgenic mice were randomly divided into the model group (AD group) and the cognitive training group (Cog-group), with 8 mice in each group, and another 8 wild-type mice were selected into the wild-type group (WT group). The Morris water maze experiment was utilized to observe the generalization and learning memory ability of each group. MRI was used to detect the functional activity and neurochemical metabolism of brain regions related to memory generalization, including mPFC, hippocampus, and Re nucleus. The experimental operation was shown in Figure 1A.

### Cognitive Training

The cognitive training method was modified based on a previous study (Talboom et al., 2014). A water T-maze was implemented to conduct cognitive training on APP/PS1 mice. A T-shaped maze was placed in a circular pool to form a water T-maze, with each arm length at 40 cm and width at 15 cm, and the water temperature at 23 ± 1°C. A circular escape platform with a diameter at 6 cm was placed 1 cm under the water surface. The reference object was placed on the inside of the cross arm. The position of the escape platform and the reference object can be freely changed in two different cross arms. The training on water T-maze includes three stages:

(1) *Adaptive training stage*: One day before training, each mouse was placed in the water T-maze from the end of the straight arm, freely explored for 3 min to familiar with the environment (no reference objects were placed).

(2) *Learning and memory stage*: The escape platform was randomly placed at the end of one cross arm, with a reference object in the same cross arm to guide the position of the escape platform. Each mouse was trained to find the escape platform correctly 8 times a day, training 6 days/week, lasting 2 weeks. The training is perfect if the mouse directly swims to and land on the escape platform after entering the water T-maze. If the mouse did not find the escape platform, it will be guided to the escape platform to learn for 15 s; each training interval was 10 min.

(3) *Memory flexibility training stage*: After the learning and memory stage, the escape platform was randomly placed at the end of one cross arm, with a reference object in the opposite cross arm to train memory flexibility. The training period and method are the same as the learning and memory stage. The training method is shown in Figure 1B.

### Memory Generalization Test

Memory generalization allows individuals to extend previously learned movement patterns to similar environments. According to this, we examined the memory generalization ability of mice by slightly changing the color and shape of the reference objects in the Morris water maze. The Morris water maze was a circular pool with 120 cm diameter, 50 cm height, and 30 cm water depth. The water temperature was constant at 23 ± 1°C. The circular pool was divided into four quadrants (recorded as the first quadrant, the second quadrant, the third quadrant, and the fourth quadrant) and an escape platform with a diameter of 6 cm was placed 2 cm under the water surface in the third quadrant. The water maze was surrounded by blue curtains, and the background was stable. The only reference objects were located on the inner wall of the water maze. A camera was mounted above the water maze to record the trajectory of each mouse. The Morris water maze experiment included 3 stages:

(1) *Positioning and navigation experiment*: At this stage, each mouse was put into the water from different quadrants. The time when each mouse entered the escape platform was recorded. If the mouse found the platform within 90 s and stayed more than 3 s, the time was recorded as the escape latency; if the mouse did not find the escape platform within 90 s, the escape latency was recorded as 90 s and the mouse was guided to the escape platform to learn for 15 s. This stage lasted for 4 days and 4 times a day.

(2) *Space exploration experiment*: On the 5th day of the experiment, the escape platform was removed. Then, each mouse was put into the water from the first quadrant, and the trajectory of each mouse within 90 s was recorded.

(3) *Memory generalization ability test*: The memory generalization ability test was performed on the 6th day of the experiment. The escape platform was placed in the first quadrant, and the shape and color of reference objects in the first quadrant and adjacent quadrants were changed. Then, each mouse was put into the water from the third quadrant, and the time that each mouse finds the escape platform was recorded as escape latency. The detection method is shown in Figure 1C.

### Magnetic Resonance Imaging

The MRI analysis system (MiniMR-60 MRI System 7.0 T) was utilized to conduct the imaging scan. At the first, the mice were induced to anesthetize with 3% isoflurane. During the imaging scan, the head of each mouse was fixed under the surface coil, and 1.5% isoflurane was used to maintain anesthesia. The body temperature of each mouse was maintained at around 37°C by a hot water circulation system, and the breathing and heart rate were monitored to ensure the life state. The MRI scan included 2 parts: functional magnetic resonance imaging (fMRI) and magnetic resonance spectroscopy (MRS).

(1) *Functional magnetic resonance imaging*: After fixing the head of the mouse, location imaging was scanned, and the position of each mouse was adjusted to ensure the head was at the center of the image. Then, the T2-weighted imaging (T2WI) technique was carried out. The scanning parameters were mentioned as: TR/TE = 4,200/35 ms, FOV = 20 × 20 mm, averages = 4, image size = 256 × 256, slices = 35, and slice thickness = 0.5 mm. After the T2-weighted imaging, the supply of isoflurane was stopped, and the fMRI was conducted when the heart rate of each mouse returned to 80 beats/min. The scanning parameters were mentioned as: Echo time = 10,279 ms, repetition time = 2,000 ms, repetitions = 200, FOV = 20 × 20 mm, averages = 4, image size = 64 × 64, slices = 30, and slice thickness = 0.5 mm. After data collection, the Statistical Parametric Mapping (SPM) and Data Processing Assistant for Resting-State fMRI (DPARSF) software packages were used for data analysis.

(2) *Magnetic resonance spectroscopy*: T2WI images were used to determine the area of interest (volumes of interest, VOI). The mPFC, hippocampus and Re nucleus were selected as the VOI for scanning, and the scanning parameters were mentioned as: TR = 1,500 ms and TE = 144 ms. The position of these neurochemicals was shown as: NAA 2.02 parts per million (ppm), Glu 2.2 ppm, and Cr 3.05 ppm. After data collection, the software package TOPSPIN (v3.1, Bruker Biospin, Germany) was used to process spectral data. A quantum estimation (QUEST) method was used to calculate the ratio of the area under the peak of NAA and Cr (NAA/Cr), which represents the neurochemical metabolism level of NAA. The neurochemical metabolism level of Glu (Glu/Cr) was calculated using the same method.

### Statistical Analysis

All data were expressed as means ± S.E.M and processed by SPSS25.0. The method of repeated measurement analysis was used in the escape latency in the Morris water maze. The data of platform crossing times, escape latency in memory generalization test and the data of MRS were analyzed by One-way ANOVA. The LSD method was used when the variance was homogeneous, and the Dunnett’s T3 test was used when the variance was uneven. *P* < 0.05 was considered statistically significant. The regional homogeneity (ReHo) value of fMRI was analyzed by the one-way ANOVA and was considered statistically significant when more than 10 clusters existed differences (*P* < 0.001). Bivariate correlation analysis was utilized to analyze the correlation between the ReHo value of mPFC, hippocampus, and memory generalization ability. Pearson coefficient was used as the correlation coefficient, and *P* < 0.05 was regarded as statistically significant.

## Results

### The Correct Rate Increased Over the Course of Cognitive Training in Alzheimer’s Disease Mice

**FIGURE 2:**
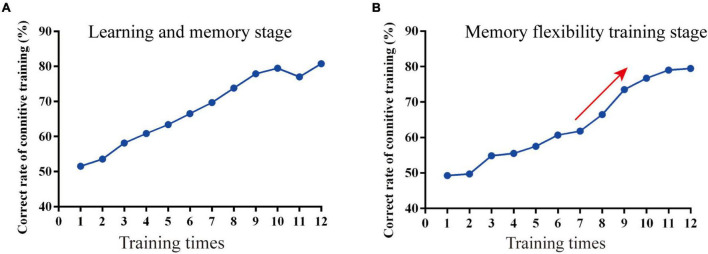
The course of the memory flexibility training in APP/PS1 mice. **(A)** The correct rate in the learning and memory stage. **(B)** The correct rate in the memory flexibility training stage.

In the learning and memory stage (phase 1), the correct rate of the mice in the Cog-group finding the escape platform was increased over time, and the correct rate was close to 80% in the last 4 days of training (Figure 2A). In the memory flexibility training stage (phase 2), the correct rate of the mice in the Cog-group finding the escape platform was increased over time. There was a rapid ascending period on the 7th day of phase 2, and the correct rate was close to 80% in the last 4 days of phase 2 (Figure 2B).

### Cognitive Training Improved the Memory Generalization Ability of Alzheimer’s Disease Mice

**FIGURE 3:**
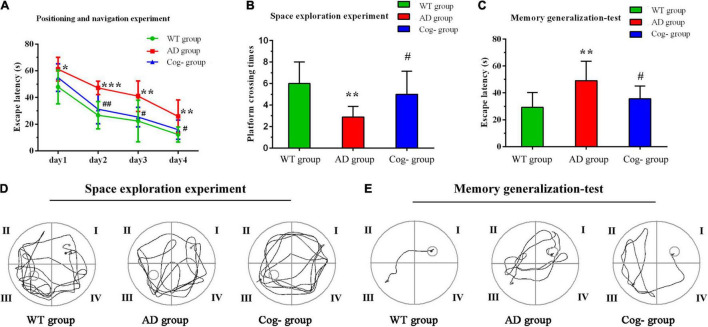
Cognition performance after cognitive training in APP/PS1 mice. **(A–E)** Learning memory and memory generalization ability after cognitive training. **(A)** The escape latency of each group in the Morris water maze. **(B)** The escape platform crossing times of each group in the Morris water maze. **(C)** The escape latency of each group in the memory generalization test. **(D)** Representative diagram of each group in the space exploration experiment. **(E)** Representative diagram of each group in the memory generalization test. *P < 0.05 compared with the WT group, **P < 0.01 compared with the WT group, ***P < 0.001 compared with the WT group, ^#^*P* < 0.05 compared with the AD group, ^##^*P* < 0.01 compared with the AD group.

The learning memory and generalization ability of each group were assessed after cognitive training. The results of Morris Water Maze showed that the escape latency of the AD group was increased compared with the WT group, while the Cog-group was decreased compared with the AD group; the escape platform crossing times of the AD group was decreased compared with the WT group, while the Cog-group was increased compared with the AD group (*P* < 0.05) (Figures 3A,B,D). After the test of Morris Water Maze, the background of the apparatus was changed to test the memory generalization ability of each group. The results showed that the escape latency of the AD group was increased compared with the WT group, while the Cog-group was decreased compared with the AD group (*P* < 0.05) (Figures 3C,E). All these results suggested that cognitive training might effectively enhance the learning memory ability of AD mice through improving the generalization ability.

### Cognitive Training Enhanced the Functional Activity of the Hippocampus and mPFC in Alzheimer’s Disease Mice

**FIGURE 4:**
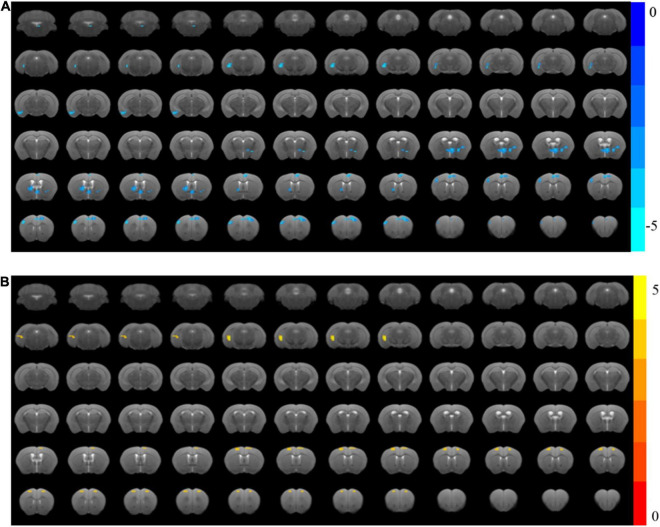
Changes in brain functional activity after cognitive training in APP/PS1 mice. **(A)** The ReHo value of the AD group decreased significantly compared with the WT group. **(B)** The ReHo value of the Cog-group increased compared with the AD group.

**FIGURE 5:**
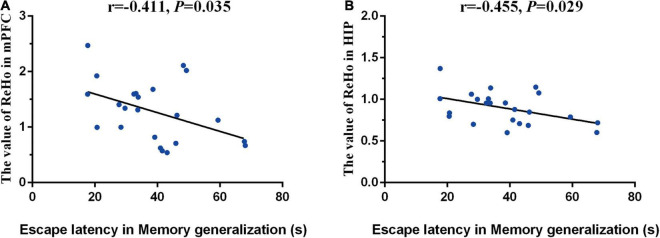
Correlations between functional activity and memory generalization ability. **(A)** Correlation between the functional activity of mPFC and memory generalization ability. **(B)** Correlation between the functional activity of hippocampus and memory generalization ability.

One mouse in the Cog-group was excluded for the head movements during MRI. MRI was completed for all 23 mice and included in the analysis of whole-brain functional activity. The results showed that compared with the WT group, the functional activity of the brain in the AD group was decreased, which included the brain regions of the hippocampus (right), mPFC, motor cortex, somatosensory cortex (right), and cingulate gyrus (right) (Figure 4A and Table 1). However, the functional activity was increased after cognitive training. As the results presented that compared with the AD group, the functional activity of the brain in Cog-group was increased, which included the brain regions of the hippocampus (right), mPFC, and motor cortex (Figure 4B and Table 1). Furthermore, the results of the Pearson’s and Spearman’s correlation analyses showed that the functional activity of the mPFC and hippocampus was all negatively correlated with the escape latency in the memory generalization test (*r* = −0.411 and −0.455, *P* < 0.05) (Figures 5A,B). These results pointed out that recovering the functional activity of the brain might be a crucial mechanism to the improvement of the memory generalization ability on AD mice.

**TABLE 1:** Regions showing significant changes in functional activity.

Brain region	AD group < WT Group		Cog-group > AD Group	
	Clusters	*t*-value	Clusters	*t*-value
Hippocampus right	15	−5.7016	13	5.2612
Medial prefrontal lobe	35	−4.5132	16	4.3032
Motor cortex left	34	−4.6837	16	4.3748
Motor cortex right	17	−4.8809	16	4.3032
Somatosensory cortex right	13	−4.8809	-	-
Cingulate gyrus right	10	−4.5209	-	-

### Cognitive Training Improved the Neurochemical Metabolism of the Hippocampus, mPFC, and Re Nucleus in Alzheimer’s Disease Mice

**FIGURE 6:**
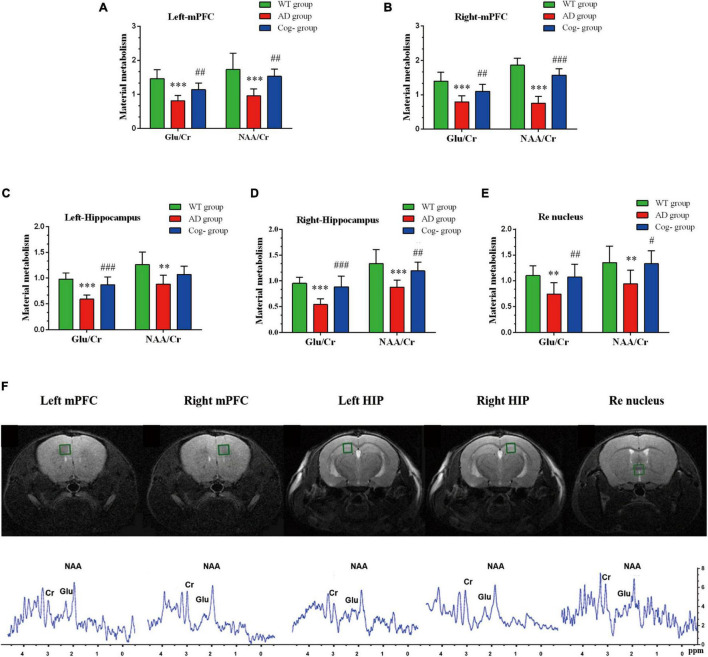
Changes in neurochemical metabolism after cognitive training in APP/PS1 mice. **(A–E)** The neurochemical metabolism of Glu and NAA of each group in mPFC (left), mPFC (right), hippocampus (left), hippocampus (right), and Re nucleus. **(F)** The location and the representative graph in mPFC (left), mPFC (right), hippocampus (left), hippocampus (right), and Re nucleus. *P < 0.05 compared with the WT group, ^**^P < 0.01 compared with the WT group, ^***^P < 0.001 compared with the WT group, ^#^*P* < 0.05 compared with the AD group, ^##^*P* < 0.01 compared with the AD group, ^###^*P* < 0.001 compared with the AD group.

In addition to functional activity analysis, MRS was performed to assess the neurochemical metabolism levels of memory generalization-related regions, including the hippocampus, mPFC and Re nucleus. In the left mPFC, the concentration of Glu and NAA in the AD group was decreased compared with the WT group (*P* < 0.001); however, the concentration of Glu and NAA in the Cog-group was increased compared with the AD group after cognitive training (*P* < 0.01) (Figure 6A). In the right mPFC, the concentration of Glu and NAA in the AD group was decreased compared with the WT group (*P* < 0.001); however, the concentration of Glu and NAA in the Cog-group was increased compared with the AD group (*P* < 0.01 or *P* < 0.001) (Figure 6B). In the left hippocampus, the concentration of Glu and NAA in the AD group was decreased compared with the WT group (*P* < 0.001 or *P* < 0.01); however, the concentration of Glu in the Cog-group was increased compared with the AD group (*P* < 0.001) (Figure 6C). In the right hippocampus, the concentration of Glu and NAA in the AD group was decreased compared with the WT group (*P* < 0.001 or *P* < 0.01); however, the concentration of Glu and NAA in the Cog-group was increased compared with the AD group (*P* < 0.001 or *P* < 0.01) (Figure 6D). In the Re nucleus, the concentration of Glu and NAA in the AD group was decreased compared with the WT group (*P* < 0.01); however, the concentration of Glu and NAA in the Cog-group was increased compared with the AD group (*P* < 0.01 or *P* < 0.05) (Figure 6E). The location of volumes of interest (VOI) and typical image of MRS are displayed in Figure 6F. All these results presented that the concentration of Glu and NAA was decreased in these brain regions related to memory generalization. Moreover, cognitive training could recover the neurochemical metabolism of AD mice.

## Discussion

Many forms of cognitive training could effectively improve cognitive function in patients with AD through changing thinking mode and memory flexibility. In this study, a T-shaped maze was carried out to conduct cognitive training on APP/PS1 mice. The results of behavior experiments presented that learning memory ability was impaired in APP/PS1 mice at 6 months of age. In contrast, cognitive training for 4 weeks could enhance the learning memory and generalization ability of APP/PS1 mice. The results of fMRI exhibited that the functional activity of mPFC and hippocampus was depressed in 6-month-old APP/PS1 mice, while cognitive training enhanced the functional activity of these two brain regions. Furthermore, the functional activity of mPFC and hippocampus was negatively correlated with the escape latency tested in the memory generalization test. At the same time, the results of MRS revealed that cognitive training was able to restore the neurochemical metabolism of Glu and NAA in mPFC, hippocampus, and Re nucleus. This study concluded that cognitive training can improve memory generalization by enhancing brain functional activity and restoring the levels of neurochemical metabolism.

The dysfunction of generalization is associated with memory deficiency in early AD. A study showed that non-dementia subjects with *ABCA7* mutation (an AD-related risk gene) are manifested as the decline of memory generalization (Sinha et al., 2019). A follow-up study indicated that subjects with poor memory generalization tests were the most likely to progress to mild cognitive impairment and Alzheimer’s disease (Myers et al., 2008). Encouragingly, clinical research has highlighted the fact that cognitive training could recover the capacity of learning memory and generalization ability in patients with cognitive decline and impairment (Chan et al., 2016; Vermeij et al., 2016). Geographical Exercise for Cognitive Optimization task training enables patients with cognition impairment to complete similar tasks effortlessly besides routine tasks (Cavallo and Angilletta, 2017). Studies in animals have also shown that cognitive training could simultaneously alleviate the impairment of memory function (Talboom et al., 2014). In this study, we performed cognitive training in APP/PS1 mice through a T-shaped water maze and trained the memory flexibility of APP/PS1 mice by changing the relative positions between the escape platform and the reference object. We found that cognition training could improve the memory generalization ability in APP/PS1 mice. The results of this study proved that the T-shaped water maze training is an effective cognitive training method to train memory flexibility. However, a recent study showed that early cognitive training could rescue the recall of remote spatial memory but reduce cognitive flexibility in AD mice (Rai et al., 2020). Such a contradiction can be attributed to the difference in the cognitive training pattern. In clinical trials, the forms of cognitive training are also multitudinous, such as television-based cognitive training, computerized cognitive training, and mathematical training (Guo et al., 2020). Effects often vary between different forms of cognitive training.

The functional connections between default networks are considered to be the neural mechanism of cognitive decline (Greicius et al., 2004; Buckner et al., 2008). Functional connections between the hippocampus and the default network are also reduced in the initial stage of AD (Dillen et al., 2017). In contrast, cognitive training could ease the symptoms of AD through enhancing the brain functional connectivity and the functional activity (Engvig et al., 2014; Lin et al., 2014). Studies have found that computer-assisted cognitive training could strengthen the functional connectivity of the hippocampus and cerebral cortex (Lin et al., 2014). A randomized controlled study also found that the blood oxygen level-dependent signals in the hippocampus and cerebral cortex were enhanced in patients with mild cognitive impairment after cognitive training (Hampstead et al., 2012). The neural circuit of mPFC-Re-hippocampus coordinates the memory information between mPFC and hippocampus, affecting memory generalization (Xu and Sudhof, 2013; Ito et al., 2018). In this study, three generalization-related brain regions were selected to conduct MRI scanning to evaluate the changes in functional activity. Our results showed that cognitive training can enhance the functional activity of mPFC and hippocampus. Furthermore, correlation analysis exhibited that the functional activity of the mPFC and hippocampus was negatively correlated with the escape latency in the memory generalization test. These results indicated that cognitive training may improve generalization ability by enhancing the functional activity of the mPFC and hippocampus. Animal studies also displayed that cognitive training could improve the cognitive function of AD model mice through enhancing hippocampus synaptic plasticity and promoting hippocampus neurogenesis (Jiang et al., 2015; Zeng et al., 2016). Similarly, cognitive training also promoted dopamine D1 receptor-mediated neuron activity in the mPFC of mice (Wass et al., 2013). Memory encoding and retrieval is closely related to the functional connection between the mPFC and hippocampus (Preston and Eichenbaum, 2013; Sekeres et al., 2018). The Re nucleus is anatomically connected with the mPFC and hippocampus to become an important neural network that regulates memory generalization (Cassel et al., 2013; Xu and Sudhof, 2013). The analysis results of this study showed that cognitive training can enhance the functional activity of the Re nucleus, but the voxel points extracted by the Re nucleus are less than 10, and the results need to be further improved (data not shown). In addition to the changes in the above-mentioned brain regions, the functional activity in the motor cortex of the Cog-group was also significantly enhanced. The method of cognitive training itself requiring mice swimming in the water T-maze might explain this phenomenon. Considering the influence of the exercise itself, it would be better to add a group to the experiment that only swimming without cognitive training.

As a non-invasive magnetic imaging technology, MRS has been extensively adopted for the detection of neurochemical metabolism in the brain of patients with neurodegenerative diseases (Gao and Barker, 2014; Sheikh-Bahaei et al., 2018). A particular study conducted whole-brain MRI scan on subjects with Alzheimer’s disease or healthy control, and the results manifested that the contents of most neurochemicals, including NAA/Cr, Cho/Cr, and mI/Cr, are significantly reduced in the brains of patients with AD, from posterior cingulate gyrus and thalamus to frontotemporal area and basal ganglia (Su et al., 2016). Abnormal brain neurochemical metabolism may be a considerable cause of cognitive impairment in Alzheimer’s disease, and scientists have pointed out that restoring neurochemical metabolism is of major significance to support cognitive function. Studies have presented that repetitive transcranial magnetic stimulation (rTMS) combined with cognitive training is potential to boost the NAA/Cr ratio in the prefrontal cortex of patients with Alzheimer’s disease, while the increase in NAA/Cr ratio was negatively correlated with the decline of Alzheimer’s Disease Assessment Scale (ADAS-cog) (Zhang et al., 2019). In addition, another study also revealed that memory enhancement training can reduce the choline compounds in the hippocampus of the elderly with mild cognitive impairment (Yang et al., 2016). At the same time, the increase in NAA/Cr is also a symbol of cognitive improvement of Alzheimer’s disease after treatment with donepezil and other drugs (Moon et al., 2016). The compound of NAA is a crucial indicator to reflect the functional state of neurons, while Glu is the most bountiful excitatory neurotransmitter in the central nervous system (Griffith et al., 2008; Chen et al., 2012; Shen et al., 2018). Clinical studies have demonstrated that patients with amnestic mild cognitive impairment have their concentration of glutamate in the cingulate gyrus decreased (Zeydan et al., 2017). Animal research also revealed that compared with the wild-type mice, the neurochemical metabolism of NAA and Glu in the cerebral cortex and hippocampus of APP/PS1 model mice was declined (Patel et al., 2018). Thus, the potential of cognitive training to improve learning memory and generalization capacity on APP/PS1 mice could be related to the increase of NAA and Glu concentration. The Re nucleus in the thalamus contains a large number of glutamatergic neurons, and studies have shown that the mPFC is capable of exciting the hippocampus indirectly through the excitatory glutamatergic relay in the Re nucleus (Hur and Zaborszky, 2005; Vertes et al., 2007). In recent years, studies have disclosed that cognitive training can not only strengthen the functional connections of the brain but also restore the extent of brain neurochemical metabolism (Yang et al., 2016; Zhang et al., 2019). In this study, we selected NAA/Cr as an indicator to reflect the functions of neurons and Glu/Cr as an indicator to reflect the activity of glutamatergic neurons. The results of this experiment displayed that the concentrations of NAA and Glu in the mPFC, hippocampus and Re nucleus of 6-month-old APP/PS1 model mice were all declined, while cognitive training for 4 weeks was capable of improving the metabolism of NAA and Glu. Apart from MRS, high-performance liquid chromatography (HPLC) is an advanced analytical technique that can quantitatively detect the distribution and content of neurochemicals. Thus, the change in neurochemical metabolism detected by MRS would be worthy of further quantitative validation.

## Conclusion

Cognitive training could improve the generalization ability that might be linked to enhance the functional activity of mPFC and hippocampus and improve the metabolism of NAA and Glu in APP/PS1 mice.

## Data Availability Statement

The original contributions presented in the study are included in the article/supplementary material, further inquiries can be directed to the corresponding author/s.

## Ethics Statement

The animal study was reviewed and approved by the Animal Management Committee of Fujian University of Traditional Chinese Medicine.

## Author Contributions

JT and WL contributed to the conception and design of the study and revised the manuscript. JL, LeL, YZ, YD, LC, WJ, and MY collected the data. SL, XH, LoL, and HL performed the statistical analysis. JL and LeL wrote the first draft of the manuscript. All authors contributed to manuscript revision, and read and approved the submitted version.

## Conflict of Interest

The authors declare that the research was conducted in the absence of any commercial or financial relationships that could be construed as a potential conflict of interest.

## Publisher’s Note

All claims expressed in this article are solely those of the authors and do not necessarily represent those of their affiliated organizations, or those of the publisher, the editors and the reviewers. Any product that may be evaluated in this article, or claim that may be made by its manufacturer, is not guaranteed or endorsed by the publisher.
